# Optimization of Polymer-ECM Composite Scaffolds for Tissue Engineering: Effect of Cells and Culture Conditions on Polymeric Nanofiber Mats

**DOI:** 10.3390/jfb8010001

**Published:** 2017-01-11

**Authors:** Ritu Goyal, Murat Guvendiren, Onyi Freeman, Yong Mao, Joachim Kohn

**Affiliations:** 1 New Jersey Center for Biomaterials, Rutgers, The State University of New Jersey, 145 Bevier Road, Piscataway, NJ 08854, USA; goyalr@dls.rutgers.edu (R.G.); muratg@njit.edu (M.G.); onyi.freeman@rutgers.edu (O.F.); maoy@dls.rutgers.edu (Y.M.); 2 Chemical, Biological and Pharmaceutical Engineering, New Jersey Institute of Technology, Tiernan Hall, University Heights, Newark, NJ 07102, USA

**Keywords:** composite polymer scaffold, biomaterial, extracellular matrix (ECM), matrix deposition, decellularization, biodegradable polymer

## Abstract

The design of composite tissue scaffolds containing an extracellular matrix (ECM) and synthetic polymer fibers is a new approach to create bioactive scaffolds that can enhance cell function. Currently, studies investigating the effects of ECM-deposition and decellularization on polymer degradation are still lacking, as are data on optimizing the stability of the ECM-containing composite scaffolds during prolonged cell culture. In this study, we develop fibrous scaffolds using three polymer compositions, representing slow (E0000), medium (E0500), and fast (E1000) degrading materials, to investigate the stability, degradation, and mechanics of the scaffolds during ECM deposition and decellularization, and during the complete cellularization-decell-recell cycle. We report data on percent molecular weight (% Mw) retention of polymeric fiber mats, changes in scaffold stiffness, ECM deposition, and the presence of fibronectin after decellularization. We concluded that the fast degrading E1000 (Mw retention ≤ 50% after 28 days) was not sufficiently stable to allow scaffold handling after 28 days in culture, while the slow degradation of E0000 (Mw retention ≥ 80% in 28 days) did not allow deposited ECM to replace the polymer support. The scaffolds made from medium degrading E0500 (Mw retention about 60% at 28 days) allowed the gradual replacement of the polymer network with cell-derived ECM while maintaining the polymer network support. Thus, polymers with an intermediate rate of degradation, maintaining good scaffold handling properties after 28 days in culture, seem best suited for creating ECM-polymer composite scaffolds.

## 1. Introduction

Extracellular matrix (ECM) is a cell-derived fibrous network, composed of proteins and glycosaminoglycans (GAGs), which displays complex combinations of biochemical, mechanical, and topographical cues. This network controls many aspects of cell function, including cell adhesion, cell-cell communication, and cell differentiation [1]. There is a significant effort to develop biomimetic scaffolds that can mimic the nanofibrous structure of ECM produced naturally by cells in vivo [2]. Electrospinning is the most widely used technique to create fibrous scaffolds for tissue engineering and drug delivery applications, due to its low cost and ease of use [3]. Polymer fibers with a wide range of fiber diameters, from nanometer to sub-micron scale, can be fabricated using electrospinning [4]. The structural features (e.g., fiber size and orientation) of the fibrous scaffolds direct many cellular functions, including adhesion [5,6,7], morphology and cytoskeletal organization [6,8,9], migration [10], differentiation [11,12,13,14], and matrix deposition [15,16,17]. Electrospun fibrous scaffolds have been made from a wide variety of natural and synthetic polymers for biomedical applications, including vascular grafts [18], skin tissue engineering [19,20], dental and craniofacial applications [21], bone and cartilage tissue engineering [22,23,24,25], nerve repair [26], and drug delivery [20,27,28,29].

For the production of electrospun fibrous scaffolds, synthetic polymers are preferred over natural polymers, as their exact composition and chemistry are well-characterized. Synthetic polymers are generally biologically inert, lacking functional sites to interact with cells; however, they do provide physical support, permitting but not promoting, basic cell function, and are thus considered a “blank state” [30,31,32]. Natural polymers, such as collagen, gelatin, and fibrin, display inherent bioactivity, but it is difficult to control their degradation properties, and they generally display much lower mechanical strength and poor handling properties. One of the most common ways to incorporate bioactivity into synthetic polymers is to chemically tether biochemical cues to the polymer backbone. This requires the polymer to have functionalizable pendant chains or end groups [29]. A less common, but increasingly investigated approach is to use cells to deposit their native ECM onto the scaffold, followed by decellularization and the use of this ECM-polymer composite scaffold as a bioactive support for subsequent recellularization [33,34,35]. This approach (which we refer to as the cellularization-decell-recell cycle) harnesses the naturally-occurring bioactive signals from a selected cell source to modify the biological responses of subsequently seeded cell populations. For instance, scaffolds containing human osteoblast-derived ECM were shown to enhance osteogenic differentiation of human embryonic stem cells [35]. In order for cells to deposit their native ECM, the scaffold should allow cellular attachment and proliferation, which requires basic cell-adhesiveness of the scaffold. It is also imperative that the fibrous scaffolds be stable under culture conditions, in the presence of cells and during the decellularization process. This could become a problem for biodegradable polymers due to the long duration of cell culture required for uniform ECM deposition. The stability of the scaffolds is crucial, as they are required to provide structural support, but they also must degrade at a rate that facilitates cellular infiltration, matrix production, and tissue development. The decellularization process requires physical, chemical, and/or enzymatic treatments, individually or in combination, which could damage the polymer scaffold [36]. In addition, ECM-polymer composite scaffolds should be stable enough to support subsequent cell culture, following the decellularization process. Despite the increasing interest in ECM-polymer composite scaffolds, studies investigating the effects of ECM-deposition and decellularization on polymer degradation, and the stability of the ECM-polymer composite scaffolds in prolonged culture conditions are still lacking. This makes it difficult to select polymers rationally and to optimize scaffold properties. To address this issue we developed fibrous scaffolds from a model polymer system consisting of three structurally-related polymer compositions, which represent fast, medium, and slow degrading polymers. 

**Figure 1:**
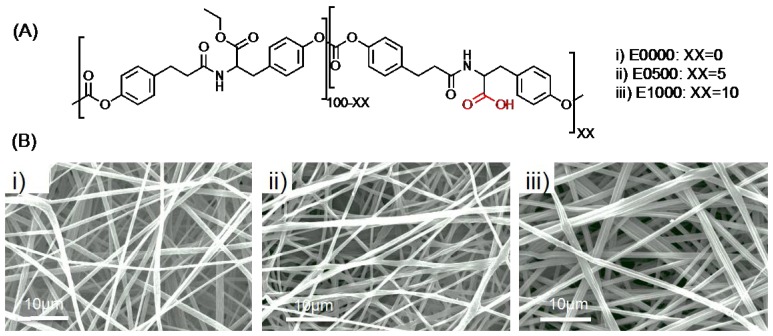
(**A**) Chemical composition of the test polymers. The free carboxylic acid group of the DT unit is marked in red for clarity. We used the notation EXX00 to name these polymers, with E denoting the ethyl ester and XX showing the mole percent of the DT. (i) E0000 denotes 0 mol% of DT; (ii) E0500 denotes 5 mol% of DT; and (iii) E1000 denotes 10 mol% percent of DT. The total amount of DT present in the polymer backbone is the only difference between the different polymers; and (**B**) SEM micrographs of electrospun fibermats made of (i) E0000; (ii) E0500; and (iii) E1000. The scaffolds had closely-matched fiber diameters and similar morphology.

Our lab was the first to develop and implement combinatorial approaches to biomaterials design [37,38,39,40]. By carefully designing monomers and synthesis routes, we have shown that it is possible to create libraries of structurally-related polymers with predictable and systematically tunable properties [37,38,41,42,43,44]. In this study, we used three polymer compositions from a previously developed library of polycarbonates as our model polymer system (Figure 1a). These polymers are composed of desaminotyrosyl-tyrosine ethyl ester (DTE), and desaminotyrosyl-tyrosine (DT) [38,44]. DTE determines strength and the modulus, whereas the carboxylic acid containing DT affects the degradation rate. The tunability of the mechanical properties and degradation rate make this library suitable for tissue engineering and drug delivery applications [45]. We used the notation EXX00 to name these polymers, with E denoting the ethyl ester and XX showing the mole percent of the DT. Previous studies of this polymer family showed that these polymers have almost identical thermal (Tg_wet_ = 35–37 °C), mechanical (tensile modulus ~1.7 ± 0.1 GPa), and water-uptake (3.2% ± 0.1%) properties up to 25% DT (E2500) [46,47]. However degradation properties changed significantly with increasing DT content. For instance, the theoretically predicted time for complete resorption in vitro of a solid pin implant was calculated to be 19.4 years for E0000 and only 3.4 years for E1000 [47]. Note that the degradation of fiber mats is expected to be much faster than the values cited above for a solid pin, due to the significantly higher surface area to volume ratio of an electrospun fiber mat. 

Three polymer compositions were used to fabricate electrospun fibrous scaffolds: E0000 (no DT present, with a slow rate of degradation), E0500 (5 mol% of DT, with an intermediate rate of degradation), and E1000 (10 mol% of DT, the fastest rate of degradation among the three test polymers). By using polymers with closely related compositions, we minimize the effect of polymer composition as an experimental parameter, allowing us to focus, in this study, on the degradation rate of the polymers. We designed stability studies, for up to 28 days, to investigate the effect of culture conditions, presence of cells, ECM deposition, decellularization process, and subsequent cell culture on the ECM-polymer composite scaffolds. 

## 2. Results and Discussion

### 2.1. Fabrication of Fibrous Scaffolds

Fibrous scaffolds (~150 microns thick) with uniform fiber diameter (~1.6 μm, with pore sizes ranging between 10 and 20 μm) were fabricated using the three test polymers: E0000, E0500, and E1000 (Figure 1). The three test scaffolds groups were identical in all aspects (fiber diameter, morphology) with the fiber composition being the only difference.

### 2.2. Stability of the Fibrous Scaffolds

**Figure 2:**
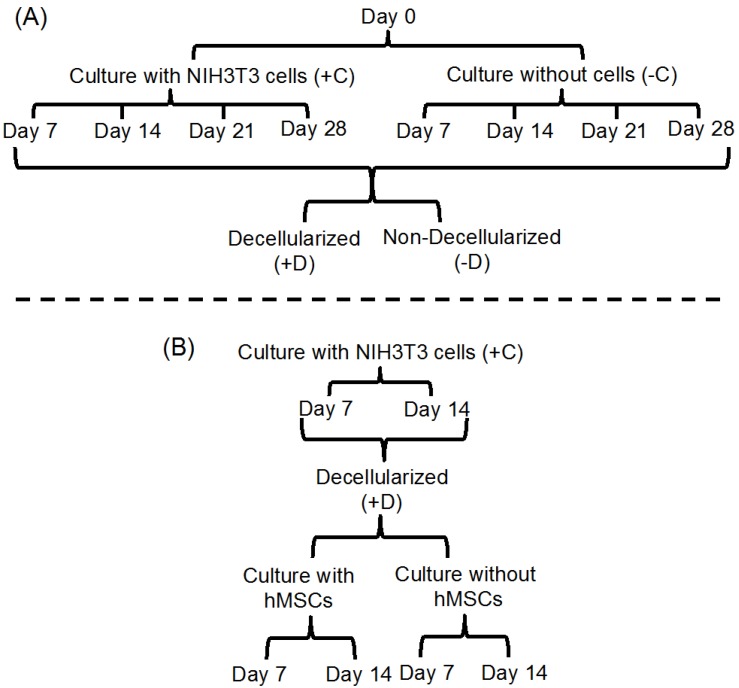
Schematic explaining the design of the stability studies. (**A**) explores the stability of fibrous scaffolds during a simple cellularization-decellularization cycle at time points from seven up to 28 days. Fibrous scaffolds were either cultured in media with NIH3T3 cells (+C) or exposed to the same incubation conditions, but without cells present (−C). The (−C) groups serve as a control and allows us to ascertain if cells affect the degradation of the polymer scaffolds. At selected time points (day 7, 14, 21, and 28), some of the scaffolds were collected and subjected to the decellularization process (+D), resulting in two treatment combinations: (+C/+D) and (−C/+D). At the same time points, some scaffolds were collected, but not subjected to the decellularization process (−D), resulting in two additional treatment combinations: (−C/−D) and (+C/−D). The (−D) groups serve as control and allow us to ascertain if the decellularization step affects the degradation of the polymer scaffolds; and (**B**) explores the full cellularization-decellularization-recellularization cycle. For days 7 and 14, the scaffolds from the (+C/+D) group were divided into two subgroups. One subgroup was cultured with hMSCs for an additional seven and 14 days to study the effect of recellularization on polymer degradation. As a control, the second subgroup was exposed to the same media conditions, but no hMSCs were present.

To investigate the stability of the fibrous scaffolds, we designed a stability study (Figure 2). In this study, NIH 3T3 cells were cultured on fibrous scaffolds (designated by “+C”) in growth medium for up to 28 days [48]. Test specimens were collected at days 4, 17, 21, and 28. At each time point the collected specimens were either decellularized (designated by “+C/+D”) or not (designated as “+C/−D”). Control experiments were also performed, in which the scaffolds were kept in growth medium in the absence of cells (designated as “−C”), and either subjected to a sham decellularization process (designated as “−C/+D”) or not (designated as “−C/−D”).

**Figure 3:**
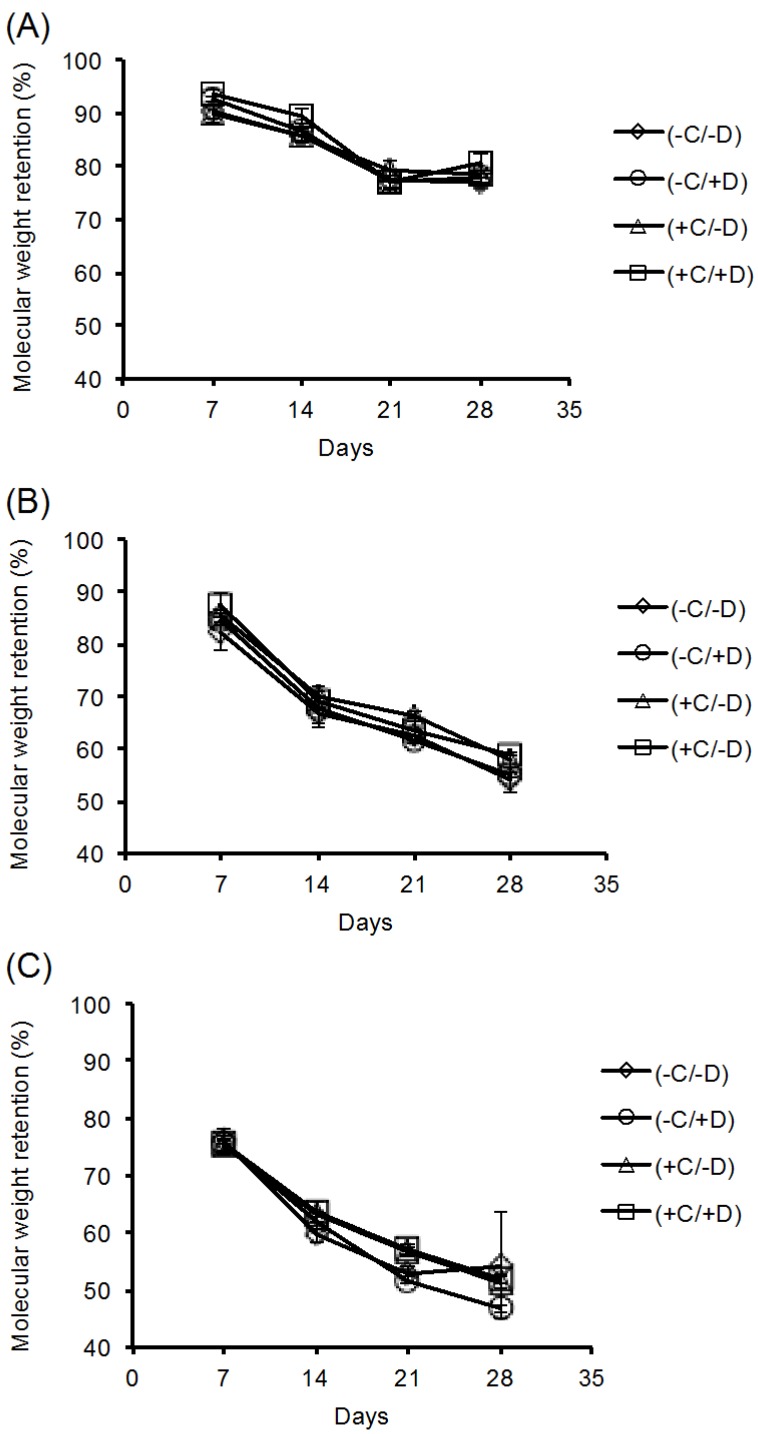
Percent molecular weight retention of (**A**) E0000; (**B**) E0500; and (**C**) E1000 scaffolds as a function of time under various culture conditions. In all graphs, error bars denote standard deviation for *n* = 3 as determined by gel permeation chromatography (GPC). While the overall rates of degradation differ for each polymer composition, data points for each of the treatment groups overlap, indicating that molecular weight retention was not affected by the presence of cells or by the decellularization process.

**Figure 4:**
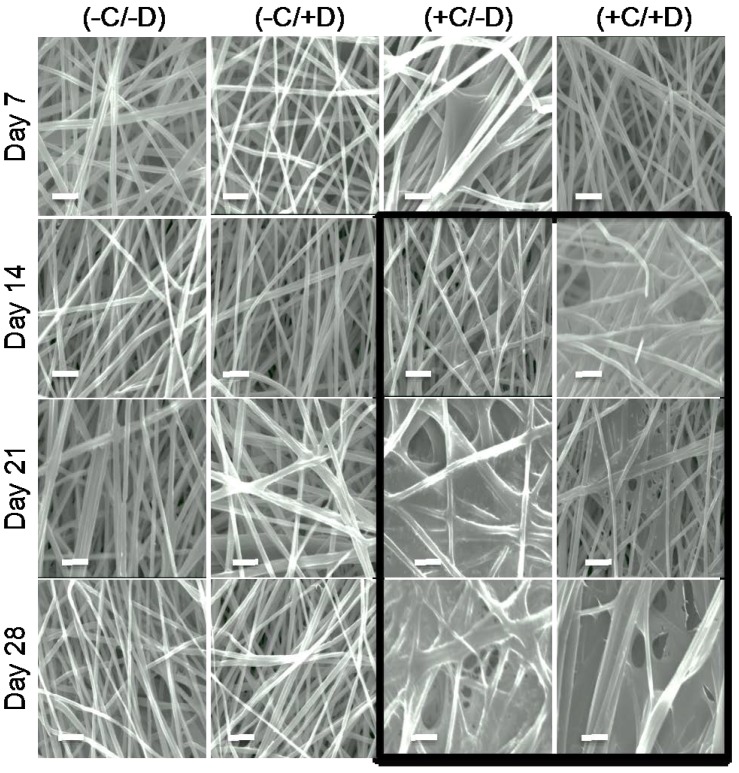
SEM micrographs of the electrospun fiber mats made of E0500 and exposed to different treatment groups for 7, 14, 21, and 28 days. Scale bars are 10 μm. The images within the black frame highlight scaffolds for which the deposition of ECM is clearly evident as a hazy coating on top of the fibers.

Polymer degradation can be mediated by hydrolysis (reaction with water that breaks the backbone bonds) or by enzyme-catalyzed reactions (biodegradation), or by cellular mechanisms following endocytosis or pinocytosis. We observed that exposing polymer scaffolds to either cell-free incubation media or cell-containing incubation media resulted in no difference in the residual polymer molecular weight after incubation. This is illustrated in Figure 3, which shows the residual molecular weight of the polymer fibers in the scaffold as function of the different treatment groups for each of the three test polymers. Specifically, the key observation is that the graphs all overlap, indicating that the presence of cells in the culture medium did not lead to a noticeable increase in polymer degradation. Therefore, we can conclude that, for these test polymers, the degradation was mediated exclusively by hydrolysis of the polymer backbone and was lacking an enzymatic or cell-mediated component. Further, the decellularization protocol used in our studies was able to remove all of the viable cells (see staining results below) without increasing the rate of polymer degradation. These results were also confirmed by the SEM images obtained from the fibrous scaffolds for each test condition. This is illustrated by Figure 4, which shows the SEM images for the E0500 scaffold under all the conditions tested (please refer to Supplementary Figures S1 and S2, for E0000 and E1000, respectively).

### 2.3. Deposition of ECM on Scaffolds Cultured in the Presence of Cells

We observed ECM deposition on the scaffolds that were cultured in the presence of cells. The amount of ECM deposition seems to increase with culture time. This is demonstrated in Figure 4 in a qualitative fashion by the increased coverage of the scaffold fibers with a hazy coating. Note that the initial ECM deposition was observed at day 14 for E0000, and at day 7 for E0500 and E1000 (Figure 4, and Supplemental Figures S1 and S2). These observations confirmed that cells deposited their ECM onto fibrous scaffolds, and that the deposited ECM was preserved during the decellularization process.

**Figure 5:**
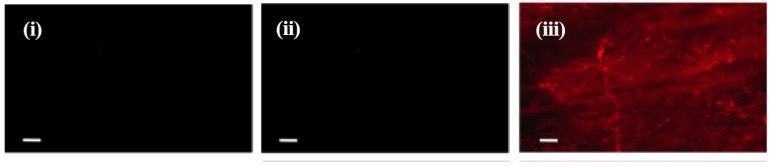
Fluorescent images of fibrous scaffolds made of E0500 immunostained for fibronectin (red). (**i**) Scaffolds were incubated in culture medium without cells for 28 days and were decellularized (−C/+D). Negative staining indicates the absence of fibronectin in the scaffolds; (**ii**) scaffolds were cultured with NIH3T3 cells for 28 days and decellularized (+C/+D) but were not immunostained for fibronectin (negative control); and (**iii**) scaffolds were cultured with NIH3T3 cells for 28 days and decellularized (+C/+D), followed by immunostaining for fibronectin. The positive staining in (iii) indicates the presence of cell-derived fibronectin on the scaffolds. Scale bars are 10 μm.

All the scaffolds that were cultured with cells for 28 days and decellularized (treatment group +C/+D) were further evaluated for the presence of fibronectin within the deposited ECM. Immunostaining confirmed the presence of fibronectin in the deposited ECM in all test scaffolds (Figure 5 and Supplemental Figure S3).

### 2.4. Change in Scaffold Stiffness as Function of Time

**Figure 6:**
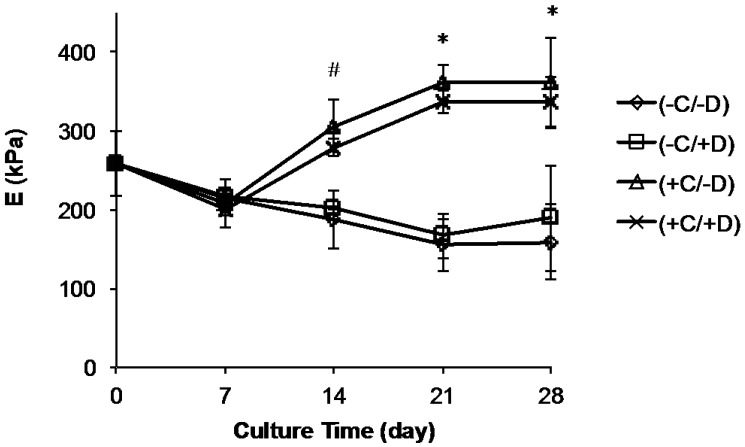
Young’s modulus (*E*) plotted against culture time for fiber mats from E0500. Starting at day 14, scaffolds cultured in the presence of cells became stiffer than scaffolds incubated without cells. ^#^ *p* < 0.1 (+C/−D) and (+C/+D) at day 14 compared to all groups at day 7, and (−C/−D) and (−C/+D) at days 14, 21, and 28; * *p* < 0.001 (+C/−D) and (+C/+D) at days 21 and 28 compared to all groups at day 7, and (−C/−D) and (−C/+D) at days 14, 21, and 28 (error bars denote standard deviation for *n* = 3 samples).

We also investigated the effect of all test conditions on the stiffness (Young’s modulus, *E*) of the fibrous scaffolds. These results are complicated by the fact that each of our test polymers had a different rate of degradation (Figure 2). For E0000 (the most stable of our test polymers), *E* did not change with culture time when scaffolds were incubated without cells (groups (−C/−D) and (−C/+D)). This is due to the fact that E0000 is so stable that it does not biodegrade significantly during the 28 day time period of our experiment. However, in the absence of cells, *E* decreased with incubation time for E0500 and E1000 since these polymers biodegrade faster and the fiber mats did lose some of their stiffness over the 28 day course of our experiment (Figure 6 and Supplementary Figure S4). 

In contrast, we observed an increase in scaffold stiffness (*E*) for all scaffolds with time when cells were cultured on fibrous scaffolds (treatment groups (+C/−D) and (+C/+D)) (Figure 6). This trend can be explained by the contribution of ECM deposition (Figure 4 and Figure 5) on the stiffness of the fibrous scaffolds. For instance, for E0500 scaffolds (+C/−D), the *E* values increased from 208 ± 15 kPa to 306 ± 32 kPa between days 7 and 14, reached 360 ± 22 kPa at day 21 and remained constant thereafter. Note that the increase in *E* was significant between days 7 and 14 for all sample groups (Supplementary Figure S4), and gradually increased further between day 14 and day 28, for all samples except E0500. Although the trend in *E* with culture time was similar for (+C/+D) and (+C/−D), there was a decrease in the *E* values for (+C/−D) due to the decellularization process when compared with (+C/+D). It seems reasonable to attribute this difference to the removal of the cells and some of the ECM during the decellularization process. 

To be useful for practical applications, it is necessary that the scaffold can be handled with ease after the cellularization-decellularization process. We, therefore, examined the handling properties of all scaffolds after 28 days in culture, in particular the ability of a scaffold to be picked up and transferred from one plate to another without breaking or tearing. We found that the scaffolds made with E1000 failed this test. It seems that for E1000, the fastest degrading polymer composition, the degree of polymer degradation was too fast to provide a sufficiently strong matrix to support the native ECM deposited by the cells. For E0000, no significant fiber degradation occurred at all, preventing the deposited ECM from replacing the polymer support. E0500 provided the optimum condition to allow gradual replacement of the polymer network with native ECM while maintaining the polymer network. Our lab has started investigating the cellular behavior of MCSs on these composite scaffolds. This work will explore the potential use of these scaffolds for tissue-specific applications, such as, skin, cartilage and ligament.

### 2.5. Testing the Full Cellularization-Decell-Recell Cycle of ECM-Polymer Composite Scaffolds

**Figure 7:**
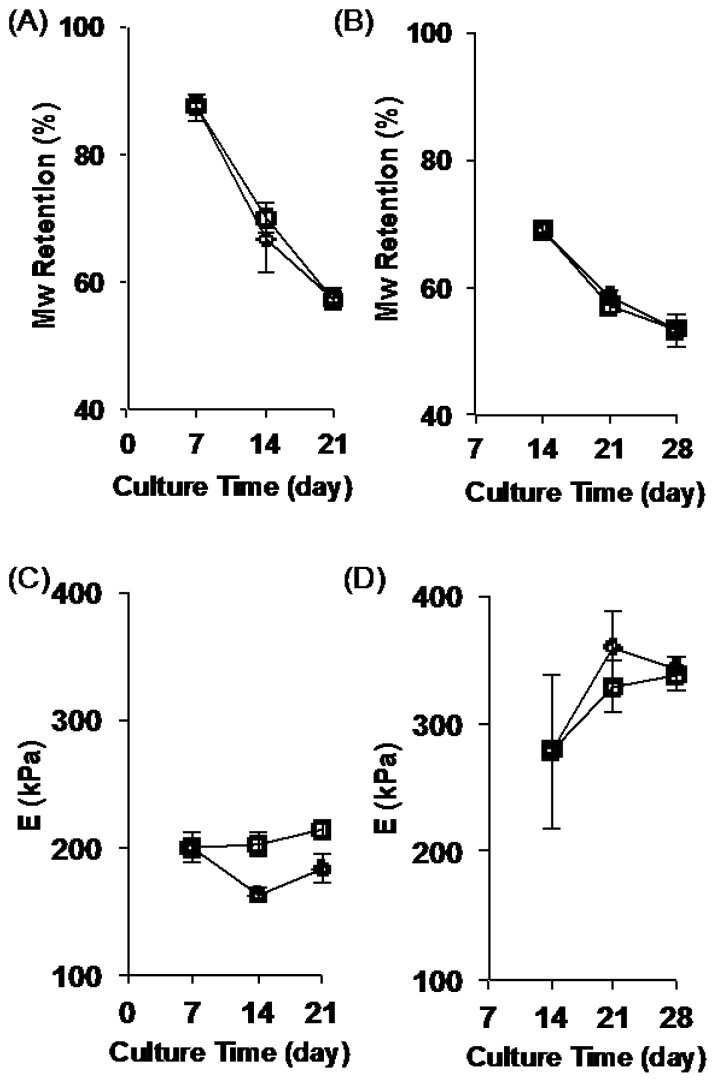
Stability of ECM-polymer composite scaffolds was tested by culturing hMSCs on these scaffolds. Percent molecular weight (% Mw) retention (**A**,**B**); and Young’s modulus (*E*) (**C**,**D**) plotted against culture time for ECM-polymer composite scaffolds from E0500 (**A**–**D**); ECM-polymer composite scaffolds were prepared by decellularizing NIH 3T3 cultured scaffolds at day 7 (**A**,**C**); or day 14 (**B**,**D**). hMSCs were then cultured on these scaffolds (squares) for additional 14 days. As a control scaffolds were incubated in the growth medium without hMSCs (diamonds). The initial values of the composite scaffolds corresponding to each sample group (day 7 for (**A**,**C**); and day 14 for (**B**,**D**)) prior to culture were also reported in the plots. Error bars denote standard deviation for *n* = 3.

To further investigate the stability of the ECM-polymer composite scaffolds, we selected scaffolds, which had been cellularized and decellularized (treatment group (+C/+D)), and then cultured human mesenchymal stem cells (hMSCs) on these scaffolds. Our results above showed that 14-day culture was enough to create ECM-polymer composite scaffolds, and because of this, we chose ECM-polymer composite scaffolds that were decellularized at day 7 and day 14 for these studies. These scaffolds were then cultured in growth media for an additional 7 or 14 days with or without hMSCs. The percent molecular weight retention of the E0500 fiber mats is shown in Figure 7A,B. The *E* values of the corresponding scaffolds for the E0500 ECM-polymer composite scaffolds cultured for an additional 7 or 14 days, with or without hMSCs, are shown in Figure 7C,D.

The values for ECM-polymer composite scaffolds (+C/−D) prior to additional culture time (day 7 or day 14) were included in each plot as a reference point. The data suggests that for ECM-polymer composite scaffolds, there is a significant decrease in the % Mw retention for E0500 and E1000 as compared to E0000 (Figure 7A,B for E0500, Supplementary Figure S5 for E0000 and E1000) after culture in growth media for additional days, independent of the presence of hMSCs. Despite the significant decrease in the % Mw retention, the mechanical properties of the scaffolds are retained during additional culture time in the presence of hMSCs (Figure 7C,D, and Supplementary Figure S5). This can be attributed to additional ECM deposition by hMSCs, which resulted in an increase in mechanical strength, particularly for fibrous scaffolds decellularized at day 14. Although we were able to collect data for scaffolds from E1000 decellularized at day 7, the scaffolds were very brittle and difficult to handle at the end of the 14 day culture period. The same composite scaffolds decellularized at day 14 completely disintegrated during the subsequent 14 day culture period. 

## 3. Materials and Methods

### 3.1. Fabrication of Fibrous Scaffolds

Polymers were synthesized using a previously published procedure [49]. The molecular weights for the polymers were 344 kDa, 487 kDa, 313 kDa and the polydispersity index was 1.95, 2.03, and 1.62, for E0000, E0500, and E1000, respectively.

### 3.2. Electrospinning

The electrospinning device consisted of a syringe pump (World Precision Instruments, Sarasota, FL, USA), high voltage DC power supply, and a rotating mandrel (Reynolds Kitchens, Lake Forest, IL, USA, 4.5 cm in diameter, rotating at ~100 rpm). E0000 was dissolved in tetrahydrofuran (THF) and *N*,*N*′-dimethylformamide (DMF) mixed solution (9:1), whereas E0500 and E1000 were dissolved in glacial acetic acid overnight at 20 ± 1 °C to prepare 15% (*w*/*v*) solutions. The polymer solution was driven using the syringe pump (10 mL, Norm-ject, Fisher Scientific, Hannover Park, IL, USA) through a 23-gauge blunt end stainless steel needle (Hamilton company, Reno, NV, USA). The flow rate was adjusted to 1 mL/h for E0000 and 0.5 mL/h for E0500, and E1000. The distance between the tip of the needle and the mandrel was set to 10 cm. A 15 kV positive electric field was applied to the gap between the needle and the grounded mandrel using the DC power supply. The fibers were collected onto a non-stick aluminum sheet wrapped around the grounded mandrel. Electrospun fibers were prepared in a class 10,000 clean room located in the New Jersey Center for Biomaterials. Fiber mats were dried under vacuum for ~12 h at 20 ± 1 °C, and stored in a vacuum desiccator. 

### 3.3. Fiber Mat Characterization

The fiber morphology was characterized by scanning electron microscope (SEM, LEO 1550 SEM, Zeiss, Thornwood, NY, USA, 20 kV, gold coated). ImageJ (National Institutes of Health, Bethesda, MD, USA) was used to measure the diameter of the fibers (at least 30 measurements from three representative images at 1000× magnification).

The molecular weight of the fiber mats was characterized by gel permeation chromatography (GPC). The GPC system consisted of a 515 high performance liquid chromatography (HPLC) pump, 717plus auto sampler, a 2414 RI detector and Empower Pro^®^ Software (Waters Corporation, Milford, MA, USA). Two PLgel columns (Polymer Laboratories, Amherst, MA, USA), 1000 and 100,000 Å were used in series. DMF containing 0.1% TFA at a flow rate of 0.8 mL/min was used as the mobile phase. The molecular weights were computed against polystyrene standards. For measurement, each sample was dried under vacuum for 12 h at 20 °C, weighed and dissolved in *N*,*N*-dimethylformamide (DMF, Sigma Aldrich Co., St. Louis, MO, USA) containing 0.1 vol% trifluoroacetic acid (TFA, Sigma Aldrich Co., St. Louis, MO, USA), and filtered with 0.45 μm polytetrafluoroethylene filter (PTFE filter, GE Healthcare Bio-Sciences, Pittsburgh, PA, USA). 

The percent molecular weight (M_w_) retention (M_w_ retention (%)) of the fibrous scaffolds was evaluated using the following equation under each culture condition:

(1) $$M_{W}\;\mathrm{retention}\;(\%)=\frac{\mathrm{Average}\;M_{W}\text{ of the polymer at the selected time point}}{\mathrm{Average}\;M_{W}\text{ of the polymer prior to the stability test}}\times 100$$

The mechanical properties of the fibrous scaffold mats were measured with a Kinexus rheometer (Malvern Instruments Inc., Westborough, MA, USA). The scaffold was compressed at a constant speed to a final normal load equal to 10 kN, immediately after an initial load of 0.01 kN was applied, to ensure the contact between the plate and the mat. The gap distance (*d*) and the normal force (FN) were used to calculate the strain (ε = Δ*h*/*h* = *d*/*h*, where h is the thickness of the mat) and the stress (σ = FN/π*r*^2^, r denotes the radius of the fiber mat). Prior to measurements, fiber mats were equilibrated in phosphate-buffered saline (1x PBS) overnight at 20 ± 1 °C.

### 3.4. Cell Culture

The mats were cut into 15 mm circles to fit into a 24-well plate, and placed onto a pre-sterile Kim wipe after weighing. Each side of the mats was sterilized under ultraviolet (UV) for 30 min. The mats were placed into 24-well plates and secured at the bottom of the wells using sterile O-rings. The mats were hydrated by adding 500 μL of sterile 1x PBS to each well, and spun at ~2200 rpm for 10 min. The mats were then incubated in sterile 1x PBS at 37 °C overnight. 1x PBS was replaced with 1 mL of Dulbecco’s modified eagle’s medium (DMEM, Sigma Aldrich Co., St. Louis, MO, USA) containing 10% fetal bovine serum (FBS) and 25 μg/mL gentamicin. Two sets of samples were prepared: fiber mats without cells (−C) and fiber mats seeded with NIH3T3 cells (passage 10; 50,000 cells/mL) (+C). The medium was changed every three days. Fiber mats were collected after 7, 14, 21, and 28 days of culture. 

### 3.5. Decellularization

The growth of cells on the fiber mats was monitored using alamarBlue assay (ThermoFisher Scientific, Hanover Park, IL, USA). At the different time points, fiber mats with or without cells were washed with 1x PBS (2 × 1 mL) after the O-rings were removed from each well. The mats were washed (2 × 1 mL) with Wash I buffer (100 mM Na_2_HPO_4_, 2 mM MgCl_2_, 2 mM EGTA; pH 9.58) and incubated at 37 °C for 15 min in 1 mL lysis buffer (8 mM Na_2_HPO_4_, 1% NP-40; pH 9.63). The lysis buffer was refreshed, and mats were incubated for an additional 45 min. The mats were then washed (2 × 1 mL) with Wash II buffer (300 mM KCl, 10 mM Na_2_HPO_4_; pH 7.53), and washed (4 × 1 mL) with H_2_O. All mats were dried under vacuum for 48 h at 20 ± 1 °C, and stored in the desiccator. 

### 3.6. Cell Culture on ECM-Polymer Composite Scaffolds

Human mesenchymal stem cells (hMSCs) were cultured on samples that had been decellularized at day 7 or day 14. For these groups, after the final water wash (during decellularization) three samples were collected and seeded with hMSCs at 10,000 cells in 1 mL (per well) of MEM alpha medium containing 10% FBS and 25 μg/mL gentamicin. Media was refreshed every three days. The hMSCs were cultured for seven or 14 days. Mats were washed (2 × 1 mL) with 1x PBS and dried under vacuum for 48 h at 20 ± 1 °C.

### 3.7. Immunostaining

Decellularized fiber mats were fixed with 4% paraformaldehyde (in 100 μL, 1x PBS) for 15 min at 20 ± 1 °C, followed by washing with 1x PBS (3 × 100 μL). Primary antibody against fibronectin (R457, Princeton University) was added (1:100 dilution, 50 μL) to each sample. To the control wells, staining buffer without primary antibody was added and samples were kept overnight for shaking followed by washing with 1x PBS (3 × 100 μL). Secondary antibody goat anti-rabbit IgG-Alexa 555 (100 μL, 1:500 dilution in staining buffer) was added to all the samples followed by incubation at 20 ± 1 °C for 2 h with shaking. The samples were washed with 1x PBS (3 × 100 μL). The scaffolds were observed under a fluorescence microscope.

### 3.8. Statistical Analysis

All values are presented as mean ± standard deviation of the mean (for *n* ≥ 3). KaleidaGraph (Synergy Software, Reading, PA, USA) was used to process the data. ANOVA with Tukey’s HSD post hoc test was performed among the groups, with significance defined as confidence level of 0.05.

## 4. Conclusions

In summary, our results support the conclusions that, for the polymers tested here, (i) cell culture conditions and decellularization do not affect polymer degradation or fiber morphology under all test conditions; (ii) polymer degradation does not affect fiber morphology, even after 28 days; and (iii) cell culture time has the greatest impact on fiber mat stability. We developed ECM-polymer composite scaffolds using three selected polymer compositions with slow (E0000), medium (E0500), and fast (E1000) rates of degradation. The molecular weight retention was found to decrease with culture time, the degree of which is determined by the degradable component (DT) in the polymer formulation. The presence or absence of cells during scaffold incubation did not result in significant changes in molecular weight retention. Fiber morphology was also maintained over 28 days. These results show that culture time was the most crucial parameter in determining the stability of the fiber mats. The amount of ECM deposition increased with culture time, which led to an increase in the stiffness of the scaffolds, even when the molecular weight of the scaffold polymer decreased. During the full cellularization-decell-recell cycle, the mechanical properties of the ECM-polymer composite scaffolds were retained during additional culture in the presence of hMSCs. However, at the end of the full cellularization-decell-recell cycle, the fast degrading polymer scaffold was very brittle and difficult to handle. This also indicated that, for the fast degrading polymer, the rate of polymer degradation limited the ability of the polymer scaffold to support sufficient native ECM deposition by the cells. For slow degrading E0000, the degradation of the scaffold was not sufficient to allow ECM deposition to replace the polymer support. Based on our results, we conclude that medium degrading E0500 provided the optimum condition to allow gradual replacement of the polymer network with deposited ECM while maintaining the polymer network support. The current study only explored the feasibility of fabrication of these ECM/polymer composite scaffolds and their stability properties during cell culture. Future work will explore the optimal mechanical properties for specific tissue types.

## Supplementary Materials

The following are available online at www.mdpi.com/2079-4983/8/1/01/s1, Figure S1: SEM images of E0000, Figure S2: SEM images of E1000, Figure S3: Immunostaining results for all polymers, Figure S4: Young’s modulus values for E0000 and E1000, Figure S5: Percent molecular weight retention and Young’s modulus values for E0000 and E1000.
