# Supplementary material for: Optimization of Polymer-ECM Composite Scaffolds for Tissue Engineering: Effect of Cells and Culture Conditions on Polymeric Nanofiber Mats
Source: J Funct Biomater. 2017 Jan 11;8(1):1. doi: 10.3390/jfb8010001 (PMC5371874; doi:10.3390/jfb8010001)
Supplement: Supplementary file 1 [file jfb-08-00001-s001.pdf]

Supplementary Materials: Development of an ECM-Polymer composite Fibrous Scaffold for Tissue Engineering Applications

Ritu Goyal, Murat Guvendiren, Onyi Freeman, Prakhar Mishra, Koustubh Dube, Yong Mao and Joachim Kohn

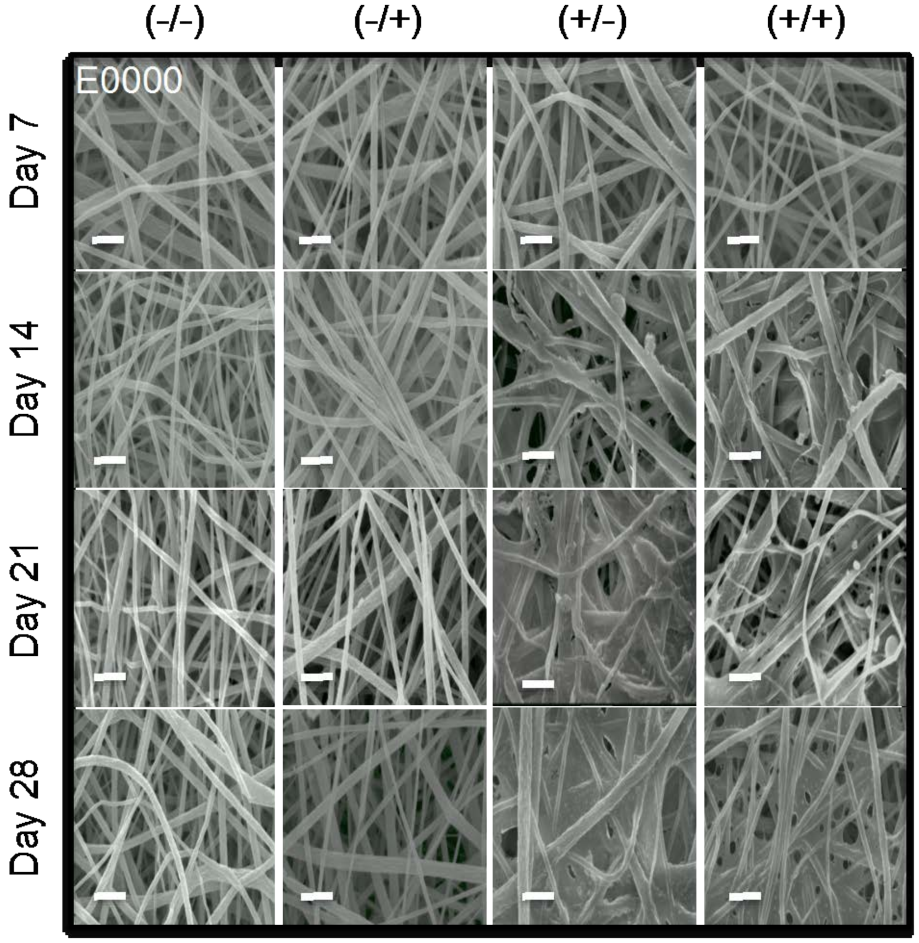

Figure S1. SEM micrographs of the electrospun scaffolds from E0000 for 7, 14, 21, and 28 days of cell culture under four conditions. Scale bars is 10 μm.

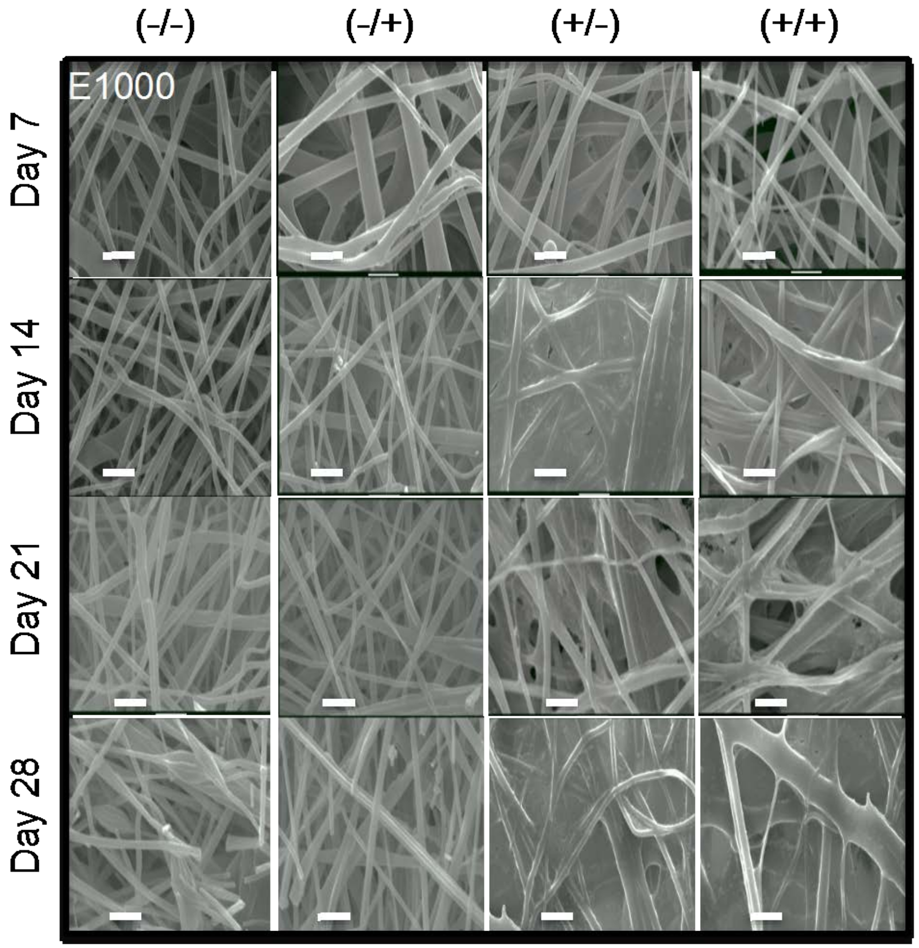

Figure S2. SEM micrographs of the electrospun scaffolds from E1000 for 7, 14, 21, and 28 days of cell culture under four conditions. Scale bars is 10 μm.

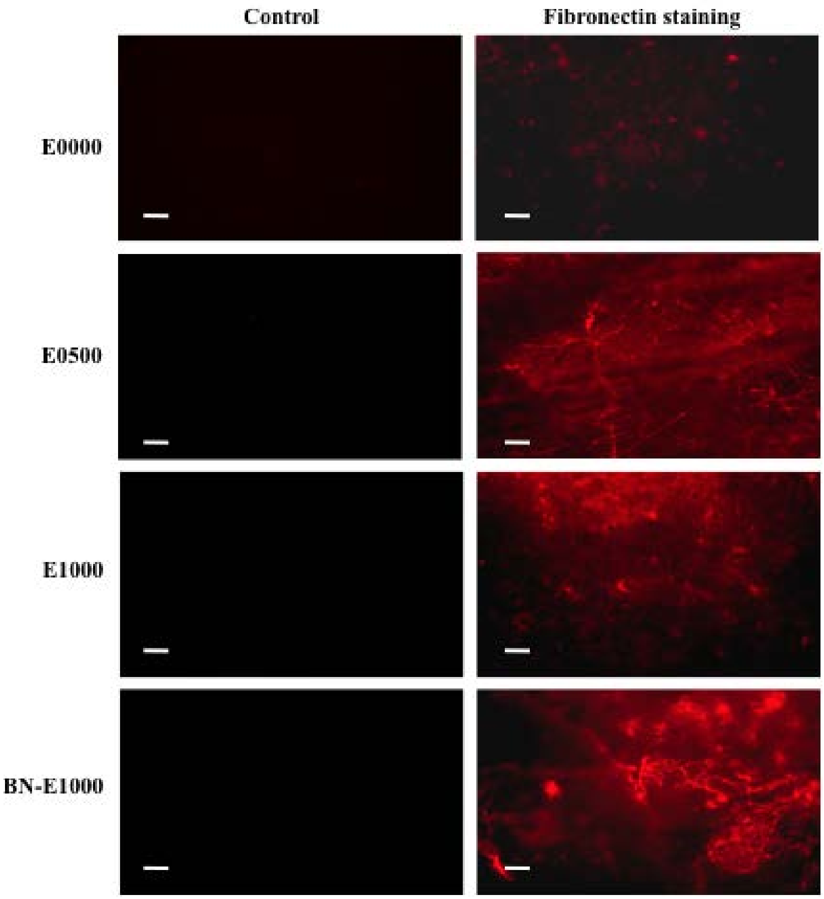

Figure S3. Immunostaining of fibrous scaffolds (+C/+D) at day 28 for all the polymers (E0000, E0500, E1000, and BN-E0500). Scale bar is 10μm.

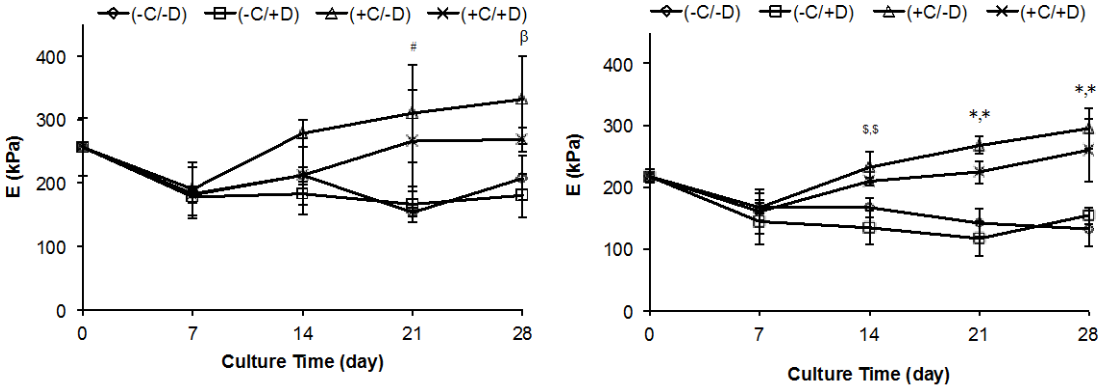

(a) (b)

Figure S4. Young's modulus (E) plotted against culture time for fiber mats from E0000 (**a**), and E1000 (**b**). $^{\square}p < 0.05$ (+C/−D) and (+C/+D) at Day 28 compared to all groups at day 7, and (−C/−D) and (−C/+D) at days 14, 21, and 28; $^{\#}p < 0.1$ (+C/−D) and (+C/+D) at Day 21 compared to all groups at day 7, and (−C/−D) and (−C/+D) at day 21 and day 28, and (−C/+D) at day 14; $^{*}p < 0.001$ and $^{\$}p < 0.01$ (+C/−D) and (+C/+D) compared to all groups at day 7, and (−C/−D) and (−C/+D) at days 14, 21, and 28 (error bars denote standard deviation for n = 3 samples).

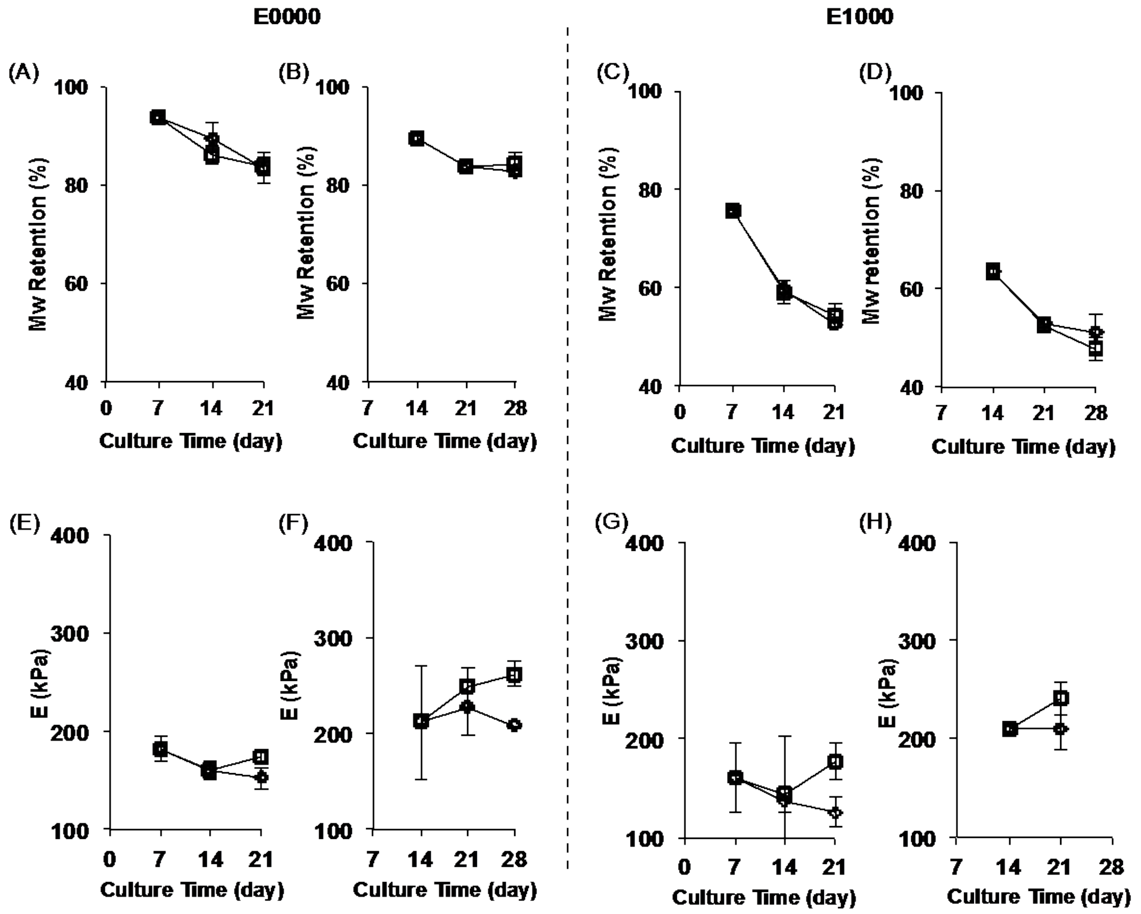

Figure S5. Percent molecular weight (Mw) retention (**A**–**D**) and Young's modulus (*E*) (**E**–**H**) plotted against culture time for decellularized fiber mats from E0000 (left panel) and E1000 (right panel). Fiber mats were cultured with NIH 3T3 cells for seven and 14 days, decellularized, and cultured with hMSCs (squares), or without hMSCs (diamonds). Error bars indicate standard deviation for n = 3.
